# Ecological insights into soil health according to the genomic traits and environment-wide associations of bacteria in agricultural soils

**DOI:** 10.1038/s43705-022-00209-1

**Published:** 2023-01-09

**Authors:** Roland C. Wilhelm, Joseph P. Amsili, Kirsten S. M. Kurtz, Harold M. van Es, Daniel H. Buckley

**Affiliations:** School of Integrative Plant Sciences, Bradfield Hall, Cornell University, Ithaca, NY 14853 USA

**Keywords:** Microbial ecology, Metagenomics, Community ecology

## Abstract

Soil microbiomes are sensitive to current and previous soil conditions, and bacterial ‘bioindicators’ of biological, physical, and chemical soil properties have considerable potential for soil health assessment. However, the lack of ecological or physiological information for most soil microorganisms limits our ability to interpret the associations of bioindicators and, thus, their utility for guiding management. We identified bioindicators of tillage intensity and twelve soil properties used to rate soil health using a 16S rRNA gene-based survey of farmland across North America. We then inferred the genomic traits of bioindicators and evaluated their environment-wide associations (EWAS) with respect to agricultural management practice, disturbance, and plant associations with 89 studies from agroecosystems. Most bioindicators were either positively correlated with biological properties (e.g., organic matter) or negatively correlated with physical and chemical properties. Higher soil health ratings corresponded with smaller genome size and higher coding density, while lower ratings corresponded with larger genomes and higher *rrn* copy number. Community-weighted genome size explained most variation in health ratings. EWAS linked prominent bioindicators with the impacts of environmental disturbances. Our findings provide ecological insights into bioindicators of soil properties relevant to soil health management, illustrating the tight coupling of microbiome and soil function.

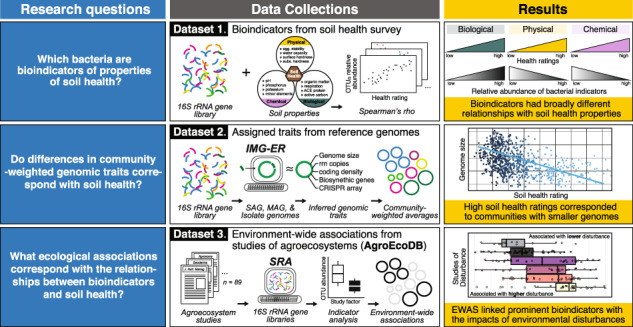

## Introduction

Managing soil health promotes the long-term fertility and ecological integrity of agricultural lands [1, 2]. Soil health encompasses a range of soil properties that contribute value to agroecosystems, including nutrient and water cycling, biodiversity, plant pathogen suppression, and pollution mitigation. Soil health is monitored using biological, physical, and chemical indicators that correspond with these functions [3–5]. Ideally, indicators should be directly linked to soil function, interpretable, and exhibit a dynamic response to management practices [6–10]. The soil microbiome has considerable potential to serve in this capacity. Microbial communities are highly sensitive to management practices [11–14], including those that shape properties that determine soil health in agricultural systems [15–20]. The broad ecological and functional diversity of bacteria in soil provides rich information about soil conditions, which was recently used to predict soil health status [21]. However, our ability to interpret the responses of bacterial ‘bioindicators’ is limited by our sparse understanding of the ecology and function of most bacteria in soil. Bridging this gap between soil microbial ecology and soil health will improve the use of microbiome data in soil health monitoring.

Ecological insight into soil microbiome structure and function can be derived by leveraging the large amounts of DNA sequencing data available in public repositories. One form of ecological inference can be derived from genomic data, whereby microbial traits can be estimated from representative genomes that are close relatives of taxa observed in phylogenetic gene marker surveys [22]. Genomic traits, such as genome size, codon usage bias, and *rrn* copy number, can be used to derive ecological information from trends in soil microbiome composition [23, 24] based on the evolutionary tradeoffs between growth, survival, and reproduction shaping these traits [25–27]. Genomic traits form the basis of several life-history frameworks that group bacteria by ecological strategies (e.g., ‘generalist’ vs. ‘specialist’) [28]; adaptive tradeoffs between growth rate, yield, and stress tolerance [26, 29, 30]; or metabolic dependency (e.g., ‘prototrophic’ vs. ‘auxotrophic’) [31]. These frameworks have been used to interpret microbiome trends associated with agricultural management practices, such as tillage intensity and nutrient management [32, 33].

While promising, the genomic inference of ecological traits has notable limitations. For example, many of the most active and abundant microorganisms in agricultural soils lack representative genomes from which traits might be predicted [34–37]. Ecological information can still be derived for these non-cultivated organisms by profiling their phylogenetic gene markers across the growing number of publicly available amplicon sequencing projects [38, 39]. An ‘environment-wide association survey’ (EWAS) approach follows the principle of reverse ecology, where information is inferred from changes in the abundance and distribution of genes across sites [40], in our case the 16S rRNA phylogenetic marker gene across environmental conditions. Traditional approaches assign a trait using curated databases [41, 42], which tend to exclude uncultured or poorly characterized taxa. This is problematic since unclassified taxa are often indicative of soil properties relevant to soil health management [21, 37, 43, 44]. In contrast, EWAS requires no prior knowledge, given the capacity to obtain information for any organism with a phylogenetic gene marker present in sequencing databases [45–48]. An EWAS approach is primarily limited by the poor quality of metadata reported for most sequencing projects [49] and a historical lack of standardization in sequencing workflows. These drawbacks are partially compensated for by the sheer volume of available sequencing projects and renewed efforts to systematize data publishing will improve the efficacy of EWAS over time [50].

Our study identified and characterized bacterial bioindicators of soil properties used in soil health assessment using a large amplicon sequencing survey of farmland across North America. Our first objective was to utilize 16S rRNA gene sequencing data to identify bioindicators that correlate with twelve biological (e.g., organic matter), physical, and chemical soil properties used in soil health assessment. We focused on profiling specific bioindicator species given the relatively minor differences observed in diversity metrics reported for our dataset [21]. Our second objective was to evaluate trends in bioindicators using (i) inferred genomic traits and (ii) a 16S rRNA gene-based EWAS to understand the ecological basis for their associations with soil health. For (i), we tested whether trends in community-weighted genomic traits corresponded to variation in soil health ratings. For (ii), we explored the environment-wide associations (EWAS) of key bioindicators using a database comprised of agricultural microbiomes (derived from 89 prior studies) that included diverse metadata grouped by study factors into broad (management practice, disturbance, and plant association) and specific categories (fertilization, land-use, tillage, drought etc.). This combined approach yielded ecological information about the most abundant bioindicators of soil health and provided new perspectives on the relationships between the soil microbiome and properties related to healthy soil function.

## Methods

### Soil health and bacterial community data collection

Our primary dataset consisted of 778 soil samples sourced from farmland across the USA, representing diverse cropping systems, as part of a soil health initiative led by Cornell University and the USDA Natural Resources Conservation Service. Soils originated from 191 unique locations that differed in agricultural management practices and soil health ratings. This dataset was used in a separate study to test the accuracy of microbiome-based machine learning for predicting soil health [21]. Our study aims to identify bioindicators and explore the underlying ecological basis for their association with soil health ratings, which have yet to be examined.

The soil properties of each sample were collected using the Comprehensive Assessment of Soil Health (CASH) framework (Table S1), which uses *biological* (soil organic matter, respiration, ACE protein, and active carbon, also known as ‘permanganate oxidizable organic carbon’), *chemical* (pH, phosphorus, potassium, and minor elements), and *physical ratings* (aggregate stability, available water capacity, soil texture, and surface and sub-surface hardness) to assess soil health [7]. Tillage data was collected for most soils (*n* = 599) and was coded as ‘till’ vs. ‘no till.’ Surface and sub-surface hardness ratings were inverted so that more compacted soils corresponded with higher ratings (opposite of CASH framework); these ratings were present for a subset of samples (*n* = 309 and 292, respectively). Measurements for each soil property were transformed using a scoring function to create a normalized rating that accounts for differences in soil texture [7]. A total health score was then calculated from the unweighted mean of all twelve ratings. Perspectives on the nature of soil health assessment and health indicators continues to evolve [10]. The soil properties in the CASH framework have been used extensively to assess the impacts of soil management practices on soil function [7].

Total DNA was extracted from soils to determine bacterial community composition and was also used to estimate microbial biomass [51]. DNA was extracted using the DNeasy PowerSoil Kit, as per manufacturers recommendation (QIAGEN, Germantown, MD, USA). DNA concentration was quantified in triplicate using the Quant-iT™ PicoGreen™ dsDNA Assay Kit (Thermo Fisher Scientific, Inc., Waltham, MA, USA). Bacterial community composition was determined through amplicon sequencing of the V4 region of the 16S rRNA gene using Illumina MiSeq (2 × 250 paired-end) and dual-indexed barcoded primers (515f/806r; sequences provided in Table S2) as previously described [21, 52]. Demultiplexing, filtering and trimming, and chimera removal were performed with *QIIME2* (v. 2020.2) [53] using default parameters and trimming left and right by 5 bp. Operational taxonomic units (OTUs) were defined as amplicon sequence variants and assigned taxonomic classifications using *QIIME2* with dependencies on *DADA2* [38] and the *Silva* database (nr_v132) [54], respectively. Raw sequencing data was archived at the National Centre for Biotechnology Information (BioProject: PRJEB35975).

### Identifying bacterial ‘bioindicators’ of soil health ratings

Bacterial OTUs indicative of soil health were determined by Spearman rank correlations using the ‘rcorr’ function in the R package *Hmisc* (v. 1.34.0) [55]. Prior to correlation analyses, OTUs occurring at low frequency (fewer than 10 samples), and at low relative abundance (<0.01% of average read depth) were removed and data was normalized by sequencing depth and reported as counts per thousand reads. *p*-values were adjusted according to the Benjamini and Hochberg false discovery rate [56]. Weak correlations (*r* < |0.3| and *p*_adj_ > 0.05) were removed [57, 58]. Indicator species analyses was used to identify bioindicators for tillage intensity using the “multipatt” function in the R package *indicspecies* (v. 1.7.12) [59]. All analyses can be reproduced with scripts included in the Supplementary Data package.

### Analysis of community-weighted genomic traits

OTUs were assigned genomic trait values using representative genomes present in public databases. Genomic traits summarized by IMG-ER [60] were downloaded (March 15th, 2020) for all isolate (*n* = 68,600), single-cell amplified (*n* = 3400), and metagenome-assembled genomes (*n* = 8800). Genomic traits were selected based on prior evidence of their correlation with life-history strategies [25–27] and availability in the IMG-ER portal, namely: genome size, coding density (total length of coding regions/genome size), *rrn* operon copy number, CRISPR arrays, and biosynthetic gene clusters (BGCs). Gene abundances were normalized by genome size. OTUs were assigned a trait value iteratively based on taxonomic classification. Unclassified OTUs at the rank genus were progressively matched to averaged trait values at higher taxonomic ranks. Most OTUs were assigned a trait value (20,148/21,463; 94%) and the majority were assigned at their lowest classified taxonomic rank (58%). The community-weighted average trait values were calculated for whole bacterial communities using the weighted mean based on the relative abundance of each OTU in the community. In addition, community-weighted *rrn* abundance was re-calculated using the *rrn*DB (v. 5.6) [61], yielding results consistent with those derived from IMG-ER data.

### Environment-wide association survey (EWAS)

The EWAS of bioindicators were determined from trends in the relative abundance of identical OTUs in other 16S rRNA gene amplicon datasets from agricultural and related terrestrial environments (full details in Supplementary Methods). In brief, we compiled 89 studies totaling 14,780 individual 16S rRNA gene amplicon libraries, termed the ‘AgroEcoDB.’ Amplicon libraries were downloaded from the Short Read Archive (May 15th, 2020) for BioProjects with taxonomic IDs for “soil metagenome” (taxID: 410658), “compost metagenome” (702656), “decomposition metagenome” (1897463), “fertilizer metagenome” (1765030), “manure metagenome” (1792145), “rhizosphere metagenome” (939928), and “wood decay metagenome” (1593443). The final database was filtered from an initial 729 BioProjects to 89 based on the following criteria: (i) common overlap of the V4 region of the 16S rRNA gene, (ii) experimental manipulations, when used, were typical of agricultural management, and (iii) contained at least 15 samples with well-curated metadata. Sequences were quality filtered and assigned to OTUs using the identical methods applied to our primary soil health amplicon data. Common OTUs were those with exact sequence matches (i.e., based on amplicon sequence variant IDs) after ensuring all sequences had the exact same length prior to processing with DADA2.

Indicator species analyses was then used to calculate an indicator value for all OTUs in the AgroEcoDB based on each individual study factor (‘EWAS indicators’; *p*_adj_ < 0.05). Study factors were categorized by management categories (e.g., inorganic vs. organic fertilizer and other broad strategies, like crop rotation), disturbance (tillage, drought etc.), plant association (bulk vs. rhizosphere soil), biome (grassland vs. cropland) and other minor categories (decomposition, soil depth etc.; see Table S3). The indicator value of EWAS indicators were scored as positive or negative based on whether the study factor was positively (reduced tillage, OM management, etc.) or negatively associated with soil health (Table S3). Our subsequent analyses provided a test of these assumptions. EWAS indicator values were averaged and assigned to their corresponding OTUs in the soil health dataset. Assigned indicator values were used to calculate community-weighted averages grouped by categories (i.e., management, disturbance, plant-association, biome, etc.) in the same way as genomic trait values.

### Statistical analyses

Statistics were performed using R (v. 4.0.3) [62] with dependency on the following packages: *reshape2* (v. 1.4.4), *ggplot2* (v. 3.3.2), *plyr* (v. 1.8.6) [63–65], and *phyloseq* (v. 1.34.0) [66]. Permutational multivariate analysis of variance (PERMANOVA) was performed on Bray-Curtis dissimilarity using the R package *vegan* (v. 2.5.7) with 999 permutations. PERMANOVA was repeated with 50 permutations of factor order to obtain average *R*^2^ values. The relative importance of community-weighted traits and EWAS for explaining variation in community composition was compared with *relaimpo* (v. 2.2.6) [67]. Co-occurrence networks were constructed for bacterial taxa (aggregated by genus) based on whether two genera shared a common bioindicator status for each of the twelve soil health ratings. Edges were weighted by the number of OTUs co-occurring between nodes (i.e., each genus) and bioindicators with negative and positive correlations with ratings were visualized separately. Co-occurrence networks were visualized using Gephi (v.0.9.2) [68] with network topography determined by the Yifan Hu ‘proportional’ force-directed graphing algorithm (relative strength = 2) [69].

## Results

### Relationships among soil health ratings

Biological ratings of soil health were highly interrelated and positively correlated with total health score and with aggregate stability (Fig. S1). Total health score was negatively correlated with surface and sub-surface hardness ratings (where a higher rating indicates greater compaction) and sand content. DNA yield was significantly positively correlated with total health score (*r* = 0.51; *p* < 0.001) but was heavily influenced by clay content, likely due to the absorptive effects of clay on DNA extraction reagents (Fig. S2).

### Bioindicators of soil properties and health ratings

**Fig. 1:**
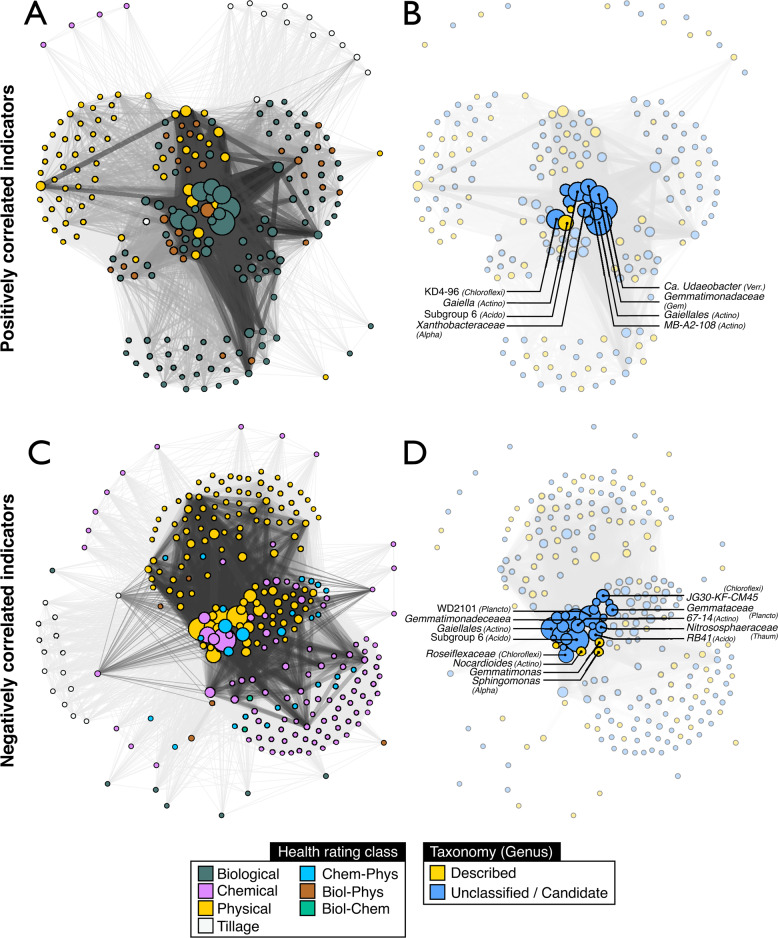
An overview of the general relationship between bioindicators of biological, physical, and chemical classes of soil properties. Among all correlated bioindicators of soil health properties shown in these co-occurrence network diagrams, the majority of positive correlations where with biological ratings (**A** and **B**), while the majority of negative correlations where with physical and chemical ratings (**C** and **D**). Networks were divided based on whether indicators exhibited positive (**A** and **B**) or negative correlations (**C** and **D**) with health ratings. In (**A**) and (**C**), nodes are colored according to health rating class and, in (**B**) and (**D**), according to whether a taxon is represented by a described species. The relationship among bioindicators was visualized in a network to highlight the prevalence of key taxa (see labeling of nodes in **B** and **D**); differences in the relationships of bioindicators with soil health classes (in **A** and **C**); and the high number of uncultured/unclassified bioindicators (in **B** and **D**). Nodes represent bioindicator OTUs aggregated to their lowest resolved taxonomic rank and scaled by the total number of OTUs. Edges represent co-occurrence of indicator OTUs for one or more of the same health rating. Edge weights are scaled by the number of co-occurring OTUs common between nodes. In (**A**) and (**C**), nodes were colored based on majority rules according to the number of OTUs representing a given health class. Classes were hyphenated when no majority was achieved.

We evaluated whether variance in OTU relative abundance was correlated with each of twelve health ratings and with total health score to identify bacterial bioindicators (*r* > |0.3| and *p*_adj_ < 0.05). A subset of OTUs (8.7%; 1874/21,463) were identified as correlated with one or more health ratings (µ = 1.5 ratings per OTU; max = 5). These ‘bioindicators’ were taxonomically diverse (348 different classifications at rank genus) with most belonging to candidate groups or unclassified genera (62%). Approximately twice as many unclassified or candidate genera (1.9-fold) were present in the bioindicator set (215/348) compared to the overall dataset (430/943). Correlations of bioindicators with biological ratings (i.e., organic matter quantity and composition) were primarily positive, while correlations with physical or chemical ratings were largely negative (Fig. 1). The majority of bioindicator OTUs were correlated in a consistent direction with one or more health ratings (96%; 1798/1874). Many genera (46%) contained a diversity of bioindicator OTUs that differed in their relationship to soil health ratings.

The main bioindicators of high biological health status were OTUs classified to Candidatus *Udaeobacter* (*Verrucomicrobia*) and *Illumatobacteraceae* (*Actinobacteria*), as well as unclassified groups of *Chloroflexi* (order *KD4-96*), *Alphaproteobacteria* (*Xanthobacteraceae*) and *Actinobacteria* (class *MB-A2-108*; full list in Table S4). The most consistent bioindicators of low physical, chemical and total health scores were OTUs classified as *Sphingomonas* (*Alphaproteobacteria*), and unclassified groups of *Chloroflexi* (order *JG30-KF-CM45*), *Archaea* (Ca. *Nitrososphaeraceae*), and *Acidobacteria* (genus *RB41*). Many highly abundant taxa (occurring at 1–5% of total read counts) were differentially abundant in tilled soils (n_OTU_ = 292) as compared to no till systems (n_OTU_ = 18; Table S4). The predominant bioindicators of tillage were members of *Alphaproteobacteria* (*Sphingomonadaceae*, *Rhizobiaceae* and *Caulobacteraceae*), *Acidobacteria* (*Pyrinomonadaceae*), *Verrucomicrobia* (genus *Chthoniobacter*) and *Actinobacteria* (genus *Terrabacter*). The main bioindicators of untilled fields coincided with the previously mentioned bioindicators of high biological health ratings, as well as *Actinobacteria* classified to *Gaiella* and unclassified groups of *Solirubrobacterales* (Table S4).

### Genomic traits linked to soil health and tillage

**Fig. 2:**
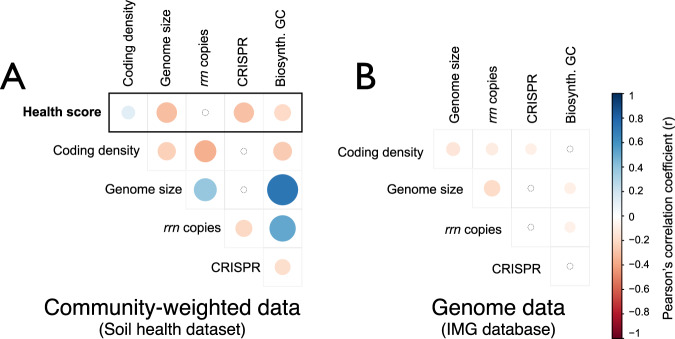
Correlations among genomic traits and also total health score. In (**A**), correlations were based on average trait scores weighted by the relative abundance of taxa-specific traits values (i.e., community-weighted data) in the soil health data. In (**B**), the same calculation was made from the genomic database used to assign trait values to taxa. This side-by-side comparison illustrates that the relationships among traits in community-weighted data partially reflected the existing relationships observed in the genomic data (Mantel statistic *r* = 0.64; *p* = 0.01). The strength of each Pearson’s correlation corresponds with color intensity as indicated by the scale provided. Circle area corresponds to the inverse of *p* value with non-significant values indicated by a small, colorless circle.

We evaluated whether community-weighted genomic traits (see Methods) explained variance in soil health ratings. Several inferred genomic traits correlated with soil health ratings. Community-weighted genome size, CRISPR array frequency, and number of BGCs were all negatively correlated with total health scores (Fig. 2A). The relationships among traits in community-weighted data broadly reflected the existing correlations among genomic traits (Mantel statistic *r* = 0.64; *p* = 0.01). However, the relationships among genome size, BGCs, and *rrn* copies in community-weighted data exhibited opposite trends from those observed in genomic databases (Fig. 2B).

**Fig. 3:**
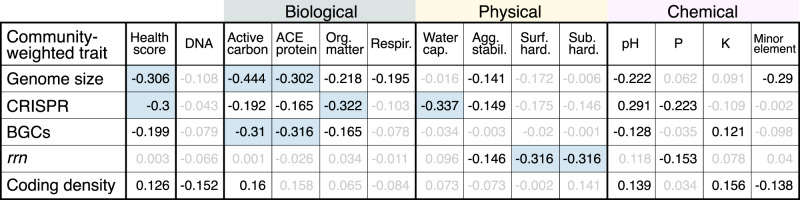
A summary of correlations between soil health ratings and community-weighted traits. Community-weighted genome size explains the most variance in overall health ratings. All Pearson’s *r* > |0.3| are shaded blue and all significant correlations are shown in bold.

Community-weighted CRISPR array frequency exhibited some of the strongest correlations with health ratings, being negatively correlated to water capacity, OM, and total health score (Fig. 3), and positively correlated with sand content (*r* = 0.44; *p* < 0.001). Community-weighted coding density was positively correlated with total health score and ratings of OM quality (active carbon and ACE protein; Fig. 3), and negatively correlated with DNA yield, a proxy for microbial biomass. The bacterial bioindicators of DNA yield with the lowest coding density were classified to *Chloroflexi* (*Ktedonobacterales*: HSB_OF53-F07 and JG30a-KF-32; µ_*density*_ = 76.2) and a family of *Planctomycetes* (*Gemmataceae*; µ_*density*_ = 79.1).

**Fig. 4:**
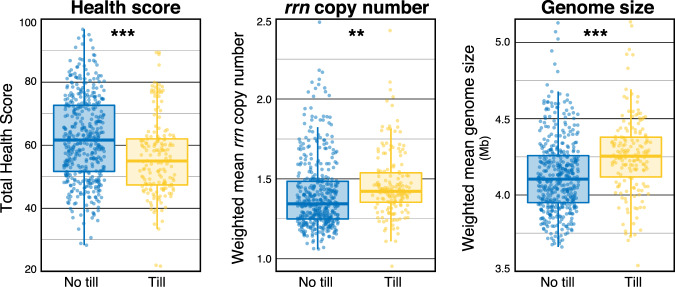
The relationship between tillage, soil health, and community weighted genomic traits. Tillage was significantly different according to variation in overall soil health rating, community-weighted *rrn* copy number, and community-weighted genome size. Significant differences are denoted with asterisk based on *t*-tests (** *p* < 0.01; *** *p* < 0.001).

Community-weighted *rrn* copy number was not correlated with total health score (*r* = 0.003), but it was significantly higher in tilled vs. untilled soils (Wilcoxon, *p* < 0.001; Fig. 4), and was correlated with surface and sub-surface hardness ratings (Fig. 3). Variance in community-weighted *rrn* copy number was driven by the abundances of *Georgenia* (µ_*rrn*_ = 5.7; *Actino*.), *Bacillaceae* (µ_*rrn*_ = 5.5; *Firmicutes*), and *Planococcaceae* (µ_*rrn*_ = 5.4; *Firmicutes*), which were all favored by tillage. Community-weighted genome size and BGC number were also higher in tilled soils relative to untilled soils (Fig. 4), and this result is consistent with their negative correlation to total health score. Community-weighted genome size and BGCs were primarily correlated with biological health ratings, unlike *rrn* copy number which was exclusively correlated with physical or chemical health ratings (Fig. 3).

### Environment-wide associations of bioindicators of soil health

The majority of OTUs in the soil health dataset were present in the AgroEcoDB (n_OTU_ = 17,818/21,573 of OTUs at 100% identity), representing a total of 96.9% of all sequences. A total of 8760 OTUs found in both datasets were identified as significant EWAS indicators (*p*_adj_ < 0.05) of one or more study factors in the AgroEcoDB. The indicator values of EWAS indicators were used to calculate community-weighted averages of broad categories and sub-categories to assess general correlations between the EWAS of bacteria and soil health. Community-weighted EWAS, inferred from amplicon data, explained more variation in bacterial community composition than community-weighted genomic traits, inferred from genomic databases (Table 1A). In contrast, community-weighted genome size explained more variation in total health score than any of the EWAS categories or sub-categories (Table 1B).

**Table 1:** Community-weighted traits and environment-wide associations (shaded) explain variation in (A) community composition according to PERMANOVA based on Bray-Curtis dissimilarity and (B) total health score according to relative importance values.

The analysis was repeated for environment-wide associations grouped at different hierarchal levels designated as category’ or ‘sub-category’ as described in Table S3.

**Fig. 5:**
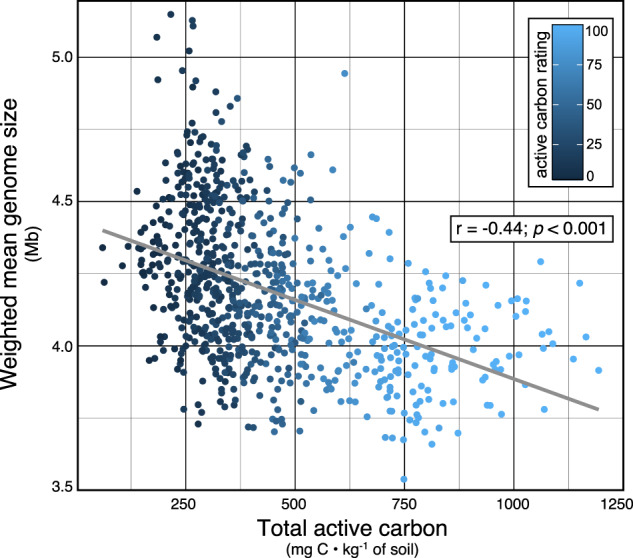
Community-weighted genome size explains significant variation in active carbon content. Points were colored on the basis of active carbon rating in soil health assessment. Differences in the proportion of unclassified taxa assigned traits was not correlated with active carbon rating (*r* = 0.06, *p* = 0.1; see Fig. S6).

**Fig. 6:**
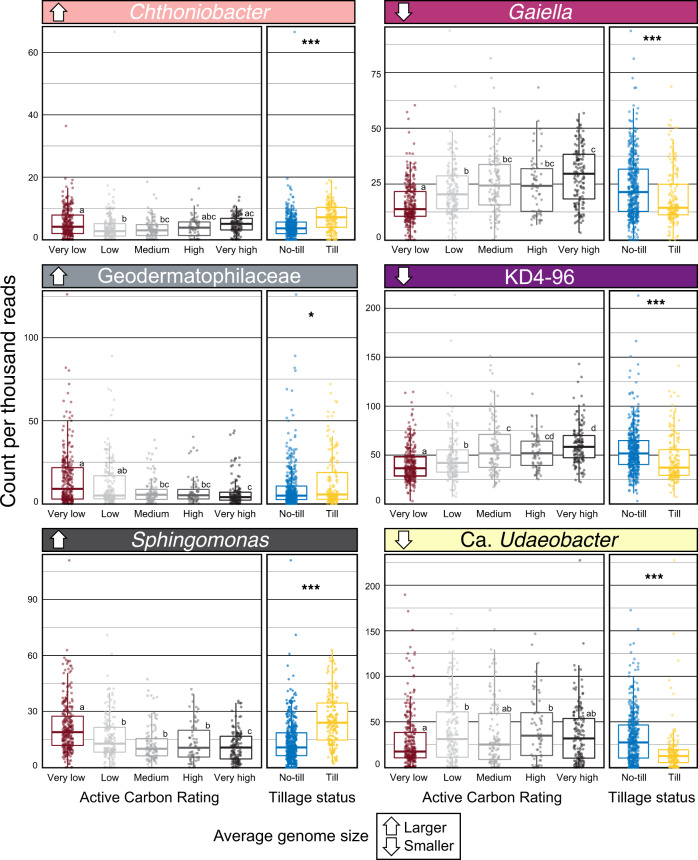
Variation in the relative abundance of six key bacterial taxa indicates that the effects of active carbon rating and tillage differ with respect to genome size. A set of six taxa were selected to represent extremes of genome size from three of the largest (left size, indicated by upward arrow), to three of the smallest (right size, indicated by down arrow). Collectively, these six taxa comprised 14% of all reads and their relative abundance has a strong impact on relationships indicated in Figs. 4 and 5. Active carbon ratings were divided into categories that range from very low (0–20) to very high (80–100). Pairwise statistically significant differences (*p* < 0.05), according to Tukey HSD, are denoted by lettering.

**Fig. 7:**
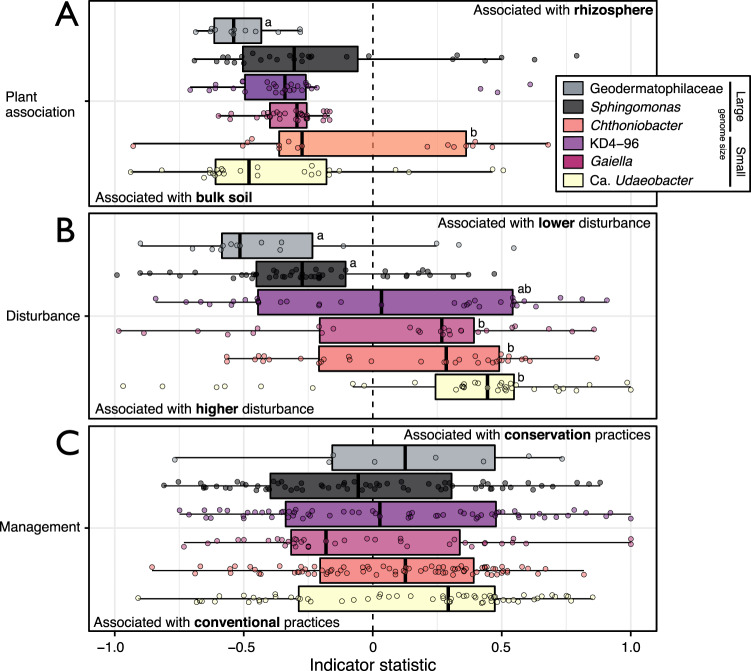
Environment-wide associations of the taxa indicated in Fig. 6. Each plot shows indicator OTUs for (**A**) plant association (i.e., bulk vs. rhizosphere soil), (**B**) soil disturbance and (**C**) management practices. In (**B**) and (**C**), indicator values were assigned as positive or negative based on whether a factor was a reference or treatment (e.g., no-till vs. till) with details of designations provided in Table S3. Only OTUs shared among the AgroEcoDB and soil health data were included. Pairwise statistically significant differences (*p* < 0.05), according to Tukey HSD, are denoted by lettering.

We examined the EWAS of the most abundant bioindicator taxa driving the relationship between community-weighted genome size and active carbon rating (Fig. 5). Active carbon rating is a measure of oxidizable soil C and it is a strong predictor of total soil health (Fig. S1). The bioindicators of active carbon with the largest estimated genome size were classified as *Chthoniobacter* (µ_size_ = 7.8 Mb; µ_rel.abund._ = 0.5% of total counts), *Geodermatophilaceae* (4.8 Mb; 1.0%), and *Sphingomonas* (4.2 Mb; 1.7%), and those with the smallest were: *Gaiella* (1.5 Mb; 2.1%), Ca. *KD4-96* (2.3 Mb; 4.9%), and Ca. *Udaeobacter* (2.7 Mb; 3.5%). Of these representative taxa, those with larger genomes had consistently higher relative abundance in soils having low active carbon ratings relative to those taxa with smaller genomes (Fig. 6). In addition, bioindicators with larger genomes were associated with tilled soils (Fig. 6). The EWAS revealed that all of these taxa were associated with bulk soil rather than rhizosphere soil (Fig. 7A). The EWAS also revealed the relationship between genome size and disturbance (Fig. 7B). These trends were driven by disturbances related to tillage (Fig. S3) and watering regimes (Fig. S4). The EWAS did not reveal any consistent effect of management practices on these representative taxa, which included crop rotation, land-use, and fertilization (Fig. 7C).

## Discussion

Our study sought to generate ecological insights into the bacterial bioindicators of biological, chemical, and physical soil properties using microbiome-based analyses of genomic traits and EWAS. Our findings provide further support for the conclusion that changes in microbiome composition are associated with variation in properties relevant to soil health management [19, 21, 32]. A diverse set of 348 bacterial genera were identified as bioindicators of one or more soil health ratings and the majority (62%) of these taxa belonged to, as yet, unclassified genera—a twofold overrepresentation compared to the whole community. This finding underscores the need for alternative, classification-independent strategies, such as genomic trait-based inference and EWAS, that allow us to gain ecological insights into uncultivated microbes from the representation of their sequences in amplicon, SAG, and MAG public databases.

### Genomic traits that correlate with soil disturbance and soil health

The relationship between community-weighted genome size and total health score was among the strongest correlations observed (Fig. 3) and was the most important predictor of variation in total health score (Table 1B). On average, communities with a greater proportion of bacteria with larger genomes occurred in soils with lower overall health rating, lower biological ratings, a history of tillage, and reduced water availability (Fig. S4). This result could indicate that soils of low health select for larger genomes and/or because they select against bacteria with smaller genomes.

Bacteria with larger genomes are hypothesized to have an advantage in habitats characterized by high environmental variability, where their expanded regulatory and metabolic capabilities allow for rapid physiological adaptation to environmental change [70, 71]. This hypothesis might suggest that large genomes are favored in soils with lower soil health because these systems exhibit more environmental instability than healthy soils, with respect to physical disturbance and moisture and nutrient availability. For example, microbes having greater metabolic flexibility are favored in tidal systems that exhibit substantial variation in moisture and nutrient availability over time [72]. Conversely, bacteria with smaller genomes often depend on interspecies interactions and community goods, and these dependencies might render them more sensitive to disturbance. For example, *Udeaobacter* possess a remarkably reduced genome [73] and they were among the strongest bioindicators of higher health ratings and exhibited a strong negative response to tillage (Figs. 6; 7; S4). *Udeaobacter* also exhibits high levels of auxotrophy and antibiotic tolerance [73, 74], which are characteristics associated with the life-history strategies exhibited by dependent organisms [31, 75].

Our analyses also illustrate the challenges of mapping the ecological characteristics of taxa on the basis of inferred genomic traits and environmental associations. For example, *Chthoniobacter* strains tended to have larger genomes (ranging from 3.6–7.8 Mb) and, consistent with other observations, were more prevalent in tilled soils (Fig. 6). However, we also found that *Chthoniobacter* were indicative of high biological ratings, which runs counter to the trends observed in other key bioindicators. The reason for this apparent contradiction remains unclear. It is possible that members of this genus occupy an as yet to be determined ecological niche that is both favored by tillage and also present in soils of high soil health. However, it is also possible that species within the genus exhibit sufficient ecological differentiation so as to be favored in different types of soils. Such niche differentiation at high phylogenetic resolution would pose a challenge to analyses that build inference from the similarity of taxonomic markers.

### Bioindicators of soil health ratings

Most bacterial taxa indicative of physical and chemical health ratings increased in relative abundance at lower soil health ratings (Fig. 1). Such relationships might be expected for stress-tolerant taxa or those that thrive under low nutrient conditions. For example, bioindicators classified to the family *Nitrososphaeraceae* were strongly indicative of soils with poor soil health ratings. *Nitrososphaeraceae* are ammonia-oxidizing archaea that have high substrate affinity and thrive under nutrient limiting conditions [76]. These taxa are commonly enriched in conventionally-managed agricultural soils fertilized with ammonia [19, 77], and they were linked to fertilizer use in our EWAS analyses (Fig. S5). It is important to note that an increase in relative abundance does not necessarily indicate an increase in absolute abundance. An increase in relative abundance does, however, indicate greater relative fitness under a given condition (i.e., potential for contributing genetic material to future generations relative to co-occurring community members), but this increase in fitness could be due to adaptations that promote prolonged survival rather than adaptations that favor reproductive growth.

Most bacterial taxa indicative of biological health ratings increased in relative abundance at higher soil health ratings. Positive correlations between bacterial relative abundance and biological ratings might indicate the enrichment of organoheterotrophs in soils with higher OM quantity and quality. Alternatively, these correlations could result from the sensitivity of these taxa to environmental disturbance, such as tillage, since demographic trends are driven by both growth and death of cells. Soils that have high health ratings tended to have high respiration and high DNA yield, suggestive of higher microbial biomass which is consistent with the hypothesis that members of the organoheterotroph community are enriched in soils with higher health ratings.

Community-weighted *rrn* copy number was significantly higher in tilled fields, which is consistent with previous findings [37]. Community-weighted *rrn* copy number was the only trait that had a significant relationship with hardness ratings and the only trait not correlated with total health score or biological health ratings (Fig. 3). Notably, hardness ratings were negatively correlated with *rrn* copy number, indicating communities in more compacted soils tended to encode a higher number of *rrn* operons. This matched our expectation that higher *rrn* copy number would be associated with more degraded soils, though the nature of the relationship remains unclear. These results confirm the influence of physical disturbance on soil bacteria, also reported by Rieke et al. [20], and their potential to serve as bioindicators of soil properties relevant to the functioning of soils.

### Exploring relationships between coding density, CRISPR array abundance, and soil health

Low coding density is a signature of obligate epibionts, endobionts, and parasites, arising from relaxed selection pressure and an accumulation of pseudogenes [78, 79]. Thus, we predicted that lower community-weighted coding density might indicate higher trophic dependency in soils supporting more microbial biomass, which correlate with higher health ratings. However, contrary to expectations, overall community-weighted coding density exhibited the opposite trend (i.e., positively correlated) with total health score. Hence, it is not clear that coding density has a straightforward relationship with soil health status.

We also explored the relationship between community-weighted CRISPR array frequency and soil health with the expectation that phage pressure would select for genomes having more arrays. CRISPR array frequency was the only genomic trait to exhibit strong inverse relationships with water capacity and OM ratings, and a positive correlation with sand content. These observations run counter to expectations that community-weighted CRISPR array abundance would be greatest in OM-rich soils, which retain moisture and would thus support higher average phage abundance [80, 81]. We hypothesize that the high community-weighted frequency of CRISPR loci in sandy soils is driven by the effects of soil texture on diffusive transport and dry-wet dynamics, which promote boom and bust predator-prey dynamics in response to episodic soil wetting events, as observed in soil biocrust communities [82]. That is, we predict that phage pressure might be best predicted from community dynamics and not community composition. However, this relationship requires further study, especially since CRISPR array frequency does not indicate the total length or number of protospacers within a given genome, which may better correlate with phage exposure [83].

## Conclusions

Genomic traits and EWAS represent relatively new strategies for exploring the ecological traits of microorganisms and both provided insight into relationships between the soil microbiome and properties relevant to soil health assessment. We show that community-weighted genome size was the best predictor of the total health rating, and this trait was also linked to tillage, active carbon, and other biological ratings. We observed a large number of bacterial taxa whose abundance was linked to tillage history, where tillage favored microbiomes with high community-weighted genome size and *rrn* copy number. Genome size is highly conserved across broad phylogenetic distances [84], lending support to our conclusions despite the fact community-weighted traits were inferred from reference genomes and at low phylogenetic resolution for many taxa. Future research is needed to confirm these observations using shotgun metagenomic approaches. Furthermore, efforts should be aimed at determining whether the bacterial bioindicators of soil health merely report on existing soil conditions or whether they underlie processes that regulate soil health. In particular, future research should focus on the relationship between genome size, carbon cycling, and soil health, since differences in genome size have been linked to differences in carbon use efficiency [85]. This relationship suggests that low health soils may select for bacteria that promote C loss, possibly causing a negative feedback that works against C accrual and restoration of degraded soils. Our study illustrates an approach for assessing the ecological attributes of bacteria linked to soil health, including unclassified and poorly characterized taxa. This kind of information is needed if we are to understand how microbiome composition is associated with agronomic management decisions that promote soil health.

## Supplementary Information

Supplementary Information

Supplementary Figures

Supplementary Tables

## Data Availability

Raw sequencing data was archived at the National Centre for Biotechnology Information (BioProject: PRJEB35975). All analyses can be reproduced with scripts included in the Supplementary Data package. The **Supplementary Data** package is available through the Open Science Foundation (10.17605/OSF.IO/UJGQF).
