# Correction: Ecological insights into soil health according to the genomic traits and environment-wide associations of bacteria in agricultural soils

**DOI:** 10.1038/s43705-023-00236-6

**Published:** 2023-04-20

**Authors:** Roland C. Wilhelm, Joseph P. Amsili, Kirsten S. M. Kurtz, Harold M. van Es, Daniel H. Buckley

**Affiliations:** School of Integrative Plant Sciences, Bradfield Hall, Cornell University, Ithaca, NY 14853 USA

**Keywords:** Microbial ecology, Metagenomics, Community ecology

Correction to: *ISME Communications* 10.1038/s43705-022-00209-1, published online 9 January 2023

The Supplementary Data package is available through the Open Science Foundation (10.17605/OSF.IO/UJGQF). The authors apologize for the oversight.

The original article has been corrected.

