# Supplementary material for: Ecological insights into soil health according to the genomic traits and environment-wide associations of bacteria in agricultural soils
Source: ISME Commun. 2023 Jan 9;3:1. doi: 10.1038/s43705-022-00209-1 (PMC9829723; doi:10.1038/s43705-022-00209-1)
Supplement: Supplementary file 1 — Supplementary Information [file 43705_2022_209_MOESM1_ESM.docx]

**Supplementary Information**

From Wilhelm *et al.*, 2022: “Ecological insights into soil health according to the genomic traits and environment-wide associations of bacteria in agricultural soils.”

*Downloading Sequencing Projects for the AgroEcoDB*

All amplicon sequencing projects were downloaded using a series of custom scripts available in the Supplementary Data package, which were executed in the following order: (1) ‘download.SRA.metagenomes’, (2) ‘define.variable.region.py’, (3) ‘download.runs.py’ and (4) ‘get.SRA.metadata.py.’ In brief, these scripts will (1) download sequencing project information for a set of provided NCBI taxonomy IDs, (2) download a subset of runs (n = 10) from each BioProject and determine which variable region of the 16S rRNA gene was targeted using HMMs from VExtractor (Hartmann et al., 2010; included in Supplementary Data) and positional overlap with a reference database, (3) download all sequencing runs from BioProjects which met the criteria (V region etc.) and, finally, (4) download associated study metadata and incorporate into a QIIME2 data object [2]. The identification of overlap with your reference database is critical for ensuring sequences are trimmed to an identical length which is necessary to obtain identical identifiers during ASV calling by DADA2 [3]. In script (3), there is code to ‘walk’ various starting positions to verify the correct information (optional; but recommended). The trimming parameters used for each sequencing project to match soil health data are provided in Table S2. The initial trimming parameters used in preparing soil health data involved removing 5 nt off of both ends of amplicons produced using the standard 515F / 805R primer pair. Script 3 should be run twice: first with option prep_trim_sheet set to ‘Y’. This will output QIIME2 summary ‘qzv’ which should be used to manually verify the sequencing quality of each project using the website: <https://view.qiime2.org/>. This will also output a ‘trim sheet’ that one can use to input trimming parameters for each project (note: trimming may yield sequence data that no longer overlaps with the reference project). In creating the AgroEcoDB, the number of reads per library was capped at 50,000, since keep all the data from larger sequencing project significantly slowed down the process. All phyloseq objects, representative sequences and indicator species output are available in the Supplementary Data package.

Several scripts for plotting EWAS output will likely be of use to readers wishing to (i) test for the associations of their own sequences (BLOCK04) and (ii) expand on the AgroEcoDB or create their own ASV-based database. However, many of the latter scripts are hardcoded in many places and do not serve as an out-of-the-box bioinformatic pipeline. There was considerable effort required to standardize metadata for downstream analyses. For example, identifying and consolidating information about the factor codes used in each experiment / sequencing project. This information is available in the Supplementary Data (‘agroecoDB - study metadata.xlsx’) but be advised that standardizing and cleaning metadata represents the greatest investment in time for building this kind of database.

*Environment-wide associations with particulate organic matter degradation*

Active carbon is positively correlated with particulate forms of soil OM (POM), such as cellulose and lignin [4, 5]. Therefore, we investigated whether the representative taxa had preferences for soluble (DOM) versus particulate forms of POM in studies utilizing soil organic matter amendments in the AgroEcoDB. *Sphingomonas* were indicators of POM amendment (Indicvalue = 0.99, *p* = 0.03) in a study comparing xylose versus cellulose soil amendment [6], while no other representatives were indicators of either DOM or POM in studies comparing amendments of cellobiose versus straw (PRJNA397131), nine substrates ranging in solubility (PRJNA668741) or a wide variety of soluble and insoluble substrates (PRJNA594403).­­­­ Representative taxa were more impacted by tillage than by the retention of plant residues in a study on soil management practices (Figure S3) [6], indicating responses to disturbance may play a greater role than functions in OM decomposition.

**References**

1. Hartmann M, Howes CG, Abarenkov K, Mohn WW, Nilsson RH. V-Xtractor : An open-source , high-throughput software tool to identify and extract hypervariable regions of small subunit ( 16 S / 18 S ) ribosomal RNA gene sequences. *J Microbiol Methods* 2010; **83**: 250–253.

2. Bolyen E, Rideout JR, Dillon MR, Bokulich NA, Abnet CC, Al-Ghalith GA, et al. Reproducible, interactive, scalable and extensible microbiome data science using QIIME 2. *Nat Biotechnol* 2019; **37**: 852–857.

3. Callahan BJ, McMurdie PJ, Rosen MJ, Han AW, Johnson AJA, Holmes SP. DADA2: High-resolution sample inference from Illumina amplicon data. *Nat Methods* 2016; **13**: 581–583.

4. Tirol-Padre A, Ladha JK. Assessing the Reliability of Permanganate-Oxidizable Carbon as an Index of Soil Labile Carbon. *Soil Sci Soc Am J* 2004; **68**: 969–978.

5. Culman SW, Snapp SS, Freeman MA, Schipanski ME, Beniston J, Lal R, et al. Permanganate Oxidizable Carbon Reflects a Processed Soil Fraction that is Sensitive to Management. *Soil Sci Soc Am J* 2012; **76**: 494–504.

6. Koechli CN. Land management affects microbial community composition and function in carbon cycling. 2016. Cornell University.
