# Supplementary material for: Ecological insights into soil health according to the genomic traits and environment-wide associations of bacteria in agricultural soils
Source: ISME Commun. 2023 Jan 9;3:1. doi: 10.1038/s43705-022-00209-1 (PMC9829723; doi:10.1038/s43705-022-00209-1)

Figure S1. Correlation plot showing pairwise Pearson's r values for all soil health metrics. Non-signifcant correlations are depicted by a hollow circle.

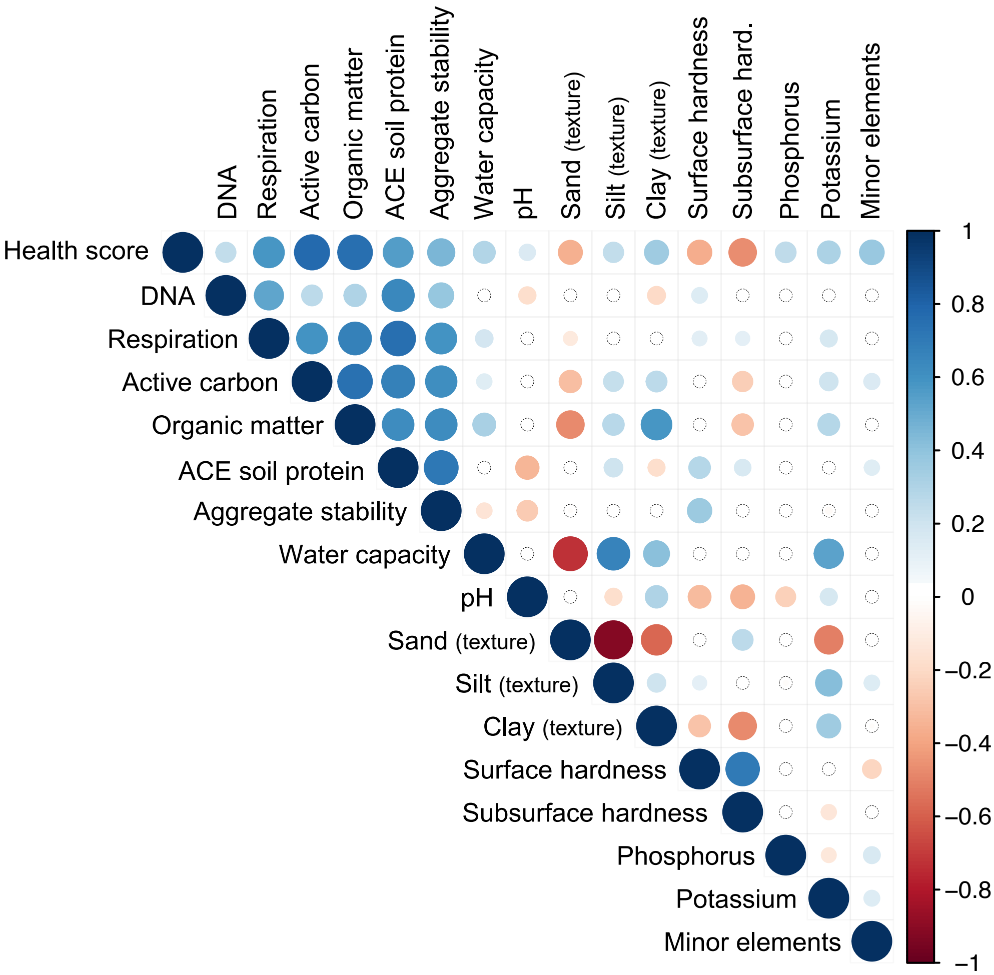

Figure S2. The correlation between soil DNA yield and total soil health rating was confounded by high clay content (A) and was improved following the exclusion of soils with > 15% clay content (B).

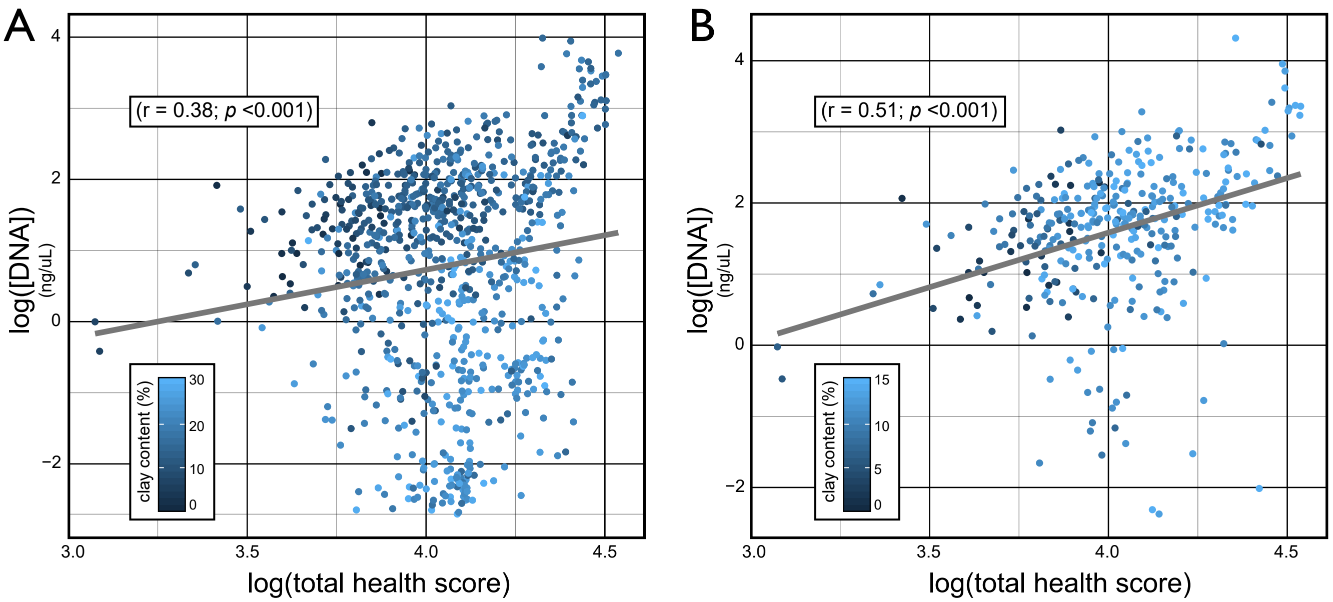

Figure S3. The association of representative taxa from either end of the spectrum of genome size with tillage status were evident in (A) their environment-wide association in the AgroEcoDB and (B) trends in relative abundance in a study of the long-term effects of tillage and plant residue retention, also present in the AgroEcoDB (Koechli, 2016). Taxa with larger genomes were found at higher relative abundances in tilled fields, while the reverse was true for taxa with smaller genomes, consistent with trends in the soil health data. In (B), statistically supported differences are denoted with lettering based on pairwise Kruskal-Wallis tests in (A) and Tukey HSD in (B) ($p < 0.05$).

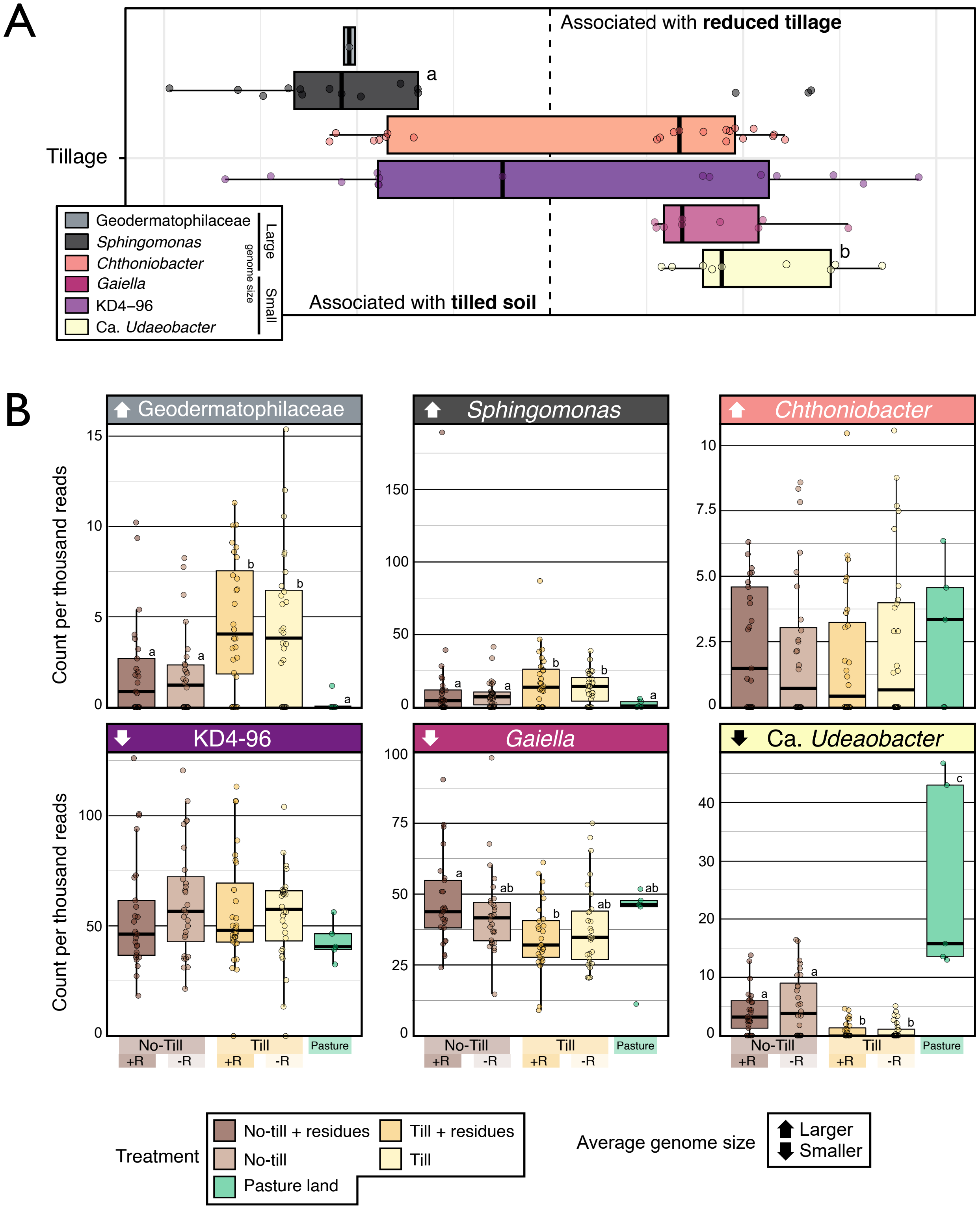

Figure S4. The association of representative taxa from either end of the spectrum of genome size with watering regime was evident in (A) their environment-wide association in the AgroEcoDB and (B) trends in relative abundance in a study of the long-term effects (40 years) of irrigation, also present in the AgroEcoDB (Azarbad et al., 2020). Taxa with smaller genomes were found at higher relative abundance irrigated fields, while the reverse was true for taxa with larger genomes. Statistically supported differences are denoted with lettering based on pairwise Kruskal-Wallis tests in (A) and Wilcoxon tests in (B; $p < 0.05$).

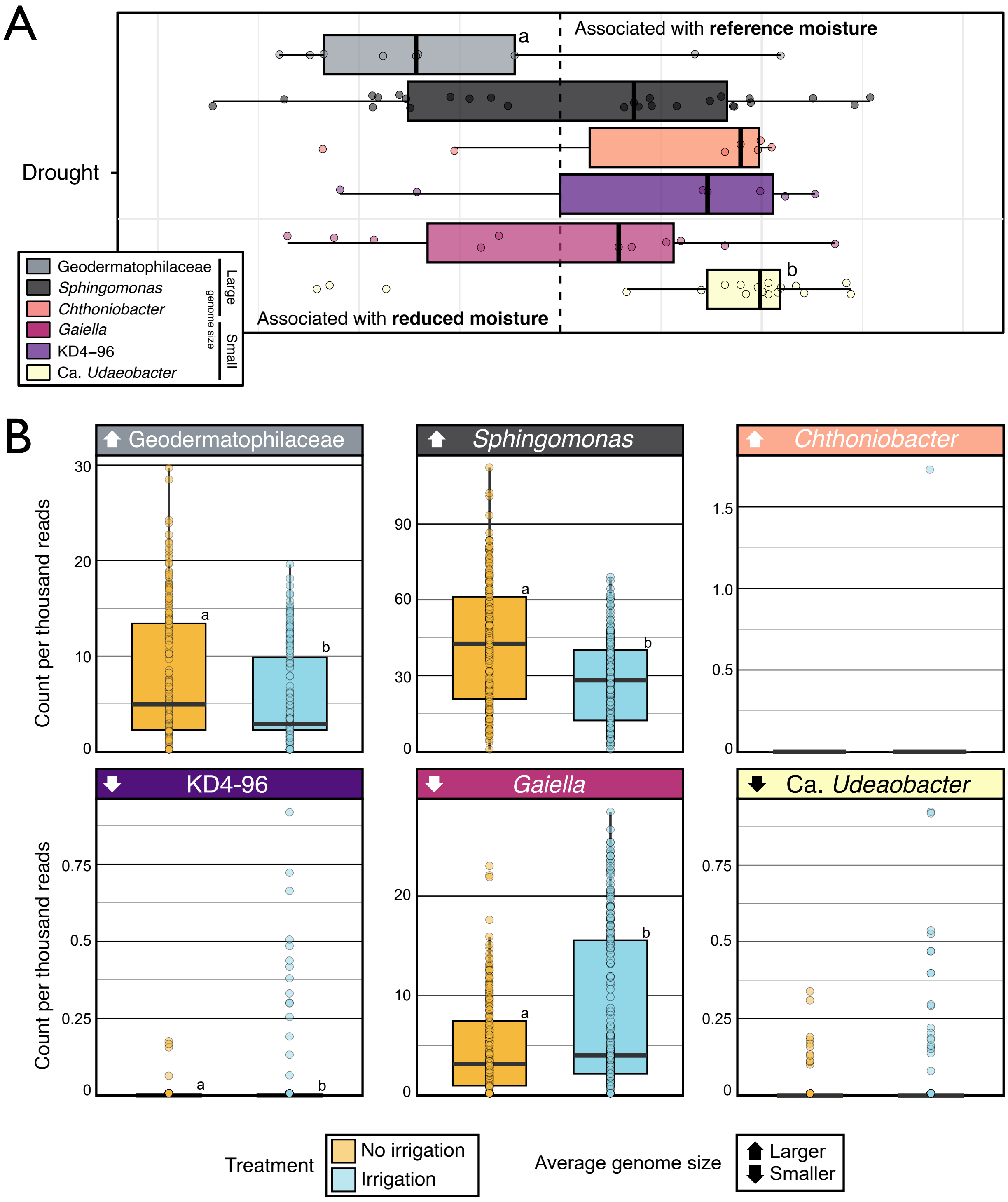

Figure S5. Environment-wide associations of *Nitrososphaeraceae* indicating an association with fertilizer use ('NPK').

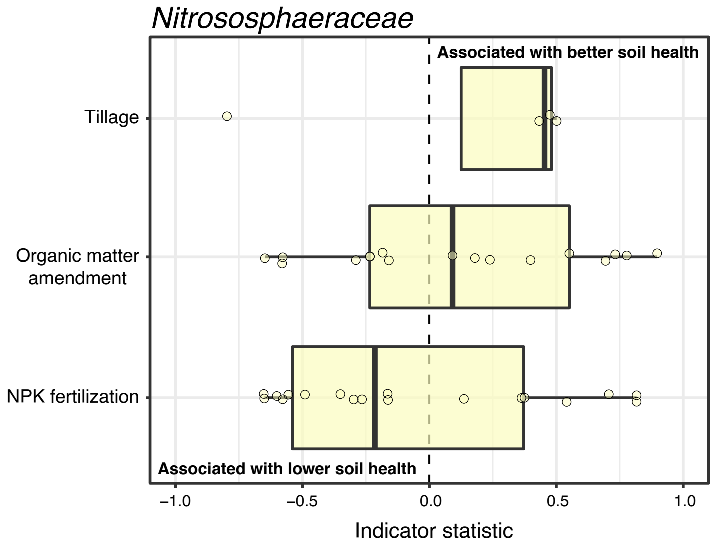

Figure S6. The relative abundance of taxa in amplicon sequencing libraries which could not be assigned a genomic trait. The y-axis corresponds to the aggregate relative abun-dance of all traitless taxa. There was no correlation with active carbon rating (r = 0.06, p = 0.1).

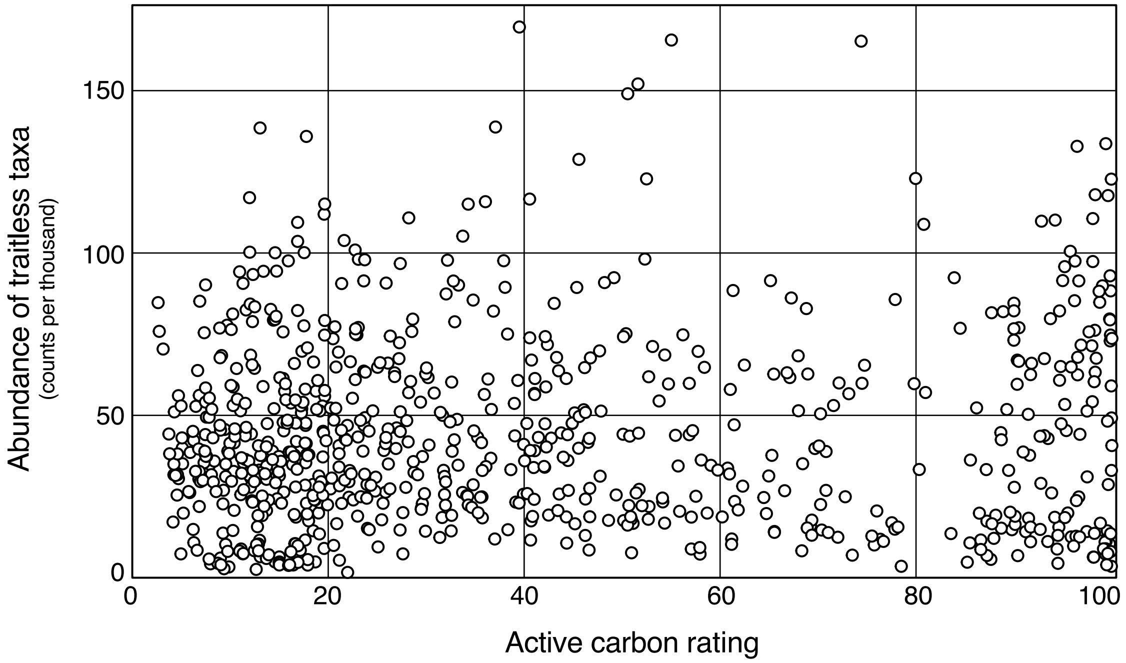

Supplement: Supplementary file 2 — Supplementary Figures [file 43705_2022_209_MOESM2_ESM.pdf]
